# Sharing Unexpected Biomarker Results with Study Participants

**DOI:** 10.1289/ehp.1001988

**Published:** 2010-09-29

**Authors:** Ann D. Hernick, M. Kathryn Brown, Susan M. Pinney, Frank M. Biro, Kathleen M. Ball, Robert L. Bornschein

**Affiliations:** 1 Breast Cancer Alliance of Greater Cincinnati, Cincinnati, Ohio, USA; 2 Department of Environmental Health, University of Cincinnati College of Medicine, Cincinnati, Ohio, USA; 3 Cincinnati Children’s Hospital Medical Center, Cincinnati, Ohio, USA

**Keywords:** biomarkers, children, communication, environmental exposures, PFOA, research ethics, transdisciplinary research

## Abstract

**Background:**

The Breast Cancer and the Environment Research Centers (BCERCs) include collaborators from basic sciences, epidemiology, and the community, conducting studies to investigate whether environmental exposures are associated with the timing of puberty. A pilot study of a subset of the study participants assessed the feasibility of measuring selected biomarkers of exposure in blood and urine in girls 6–8 years of age. In the Greater Cincinnati study population, we found an elevated serum concentration of perfluorooctanoate (PFOA) among > 90% of young girls living in a small community.

**Objectives:**

The research team deliberated whether and how to report the PFOA findings to our study families. We will address the issues considered in our decision, as well as the formats we used to present the findings.

**Methods:**

The results were verified as we searched for potential sources of the elevated PFOA levels. As a research team, we grappled with issues regarding the reporting of unexpected results, derived from unknown sources and with unknown clinical significance. Ultimately, we did decide to present these findings to the study families through a well-developed communication plan.

**Discussion:**

Research team members came from a variety of experiences and backgrounds, which led to different interpretations about the clinical, ethical, and public health issues surrounding these findings. The ethical debates centered around the precautionary principle, the right to know, and do no harm.

**Conclusions:**

Given advances in environmental biomarker technologies and greater use of the transdisciplinary research model, a communication plan must be developed for those involved as study participants.

Recent articles have discussed the obligations researchers have to inform study participants about their individual research results (Resnik 2009; Sly et al. 2009). Deciding whether and how to inform research participants about risks they face in their environment is a challenging task. The decision becomes more complicated when the research involves measuring biomarkers of exposure in a study population of children. Some ethical questions relate to children as research participants, and others are specific to biomarker research. Our experience reporting biomarker results to the families of our study participants provides a concrete example that is important to share with the research community. We present a summary of the biomarker findings of concern, how the results were reported to study families and the competing perspectives we balanced in deciding to report back unexpected results.

## Background

The University of Cincinnati (UC), in conjunction with the Cincinnati Children’s Hospital Medical Center (CCHMC) and local breast cancer advocates, forms one of four Breast Cancer and the Environment Research Centers (BCERCs) established in September 2003 with funding from the National Institute of Environmental Health Sciences and the National Cancer Institute. The BCERCs include projects in biology, epidemiology, and community outreach and education. The unifying research theme is based on the hypothesis that chemical, physical, and social factors in the environment interact with genetic factors to affect the mammary gland during peripubertal development and across the life span in ways that may alter breast cancer risk in later life. This transdisciplinary research paradigm involves biologists, epidemiologists, clinicians, and breast cancer advocates (Hiatt et al. 2009).

The specific aims of the epidemiology research are to understand more fully the impact of environmental factors on pubertal maturation in young girls and how puberty may serve as a window of susceptibility for breast cancer later in life. These objectives are predicated on the established association between age at menarche and breast cancer risk (Clavel-Chapelon 2002). Examination of environmental factors and pubertal onset could help us better understand breast cancer etiologies and inform prevention strategies.

The Greater Cincinnati epidemiology project, known as Growing Up Female (GUF), recruited 6- and 7-year-old girls and their families from school districts in southwest Ohio and across the Ohio River in northern Kentucky. Recruitment began in Spring 2004 in the two school districts. This research study was conducted with the oversight of the CCHMC Institutional Review Board (IRB), and participation of the subjects did not occur until after written consent was obtained. These early recruits were seen every 6 months at the district schools during school hours. Anthropometric measures, including height, weight, and maturation assessment, were conducted at each visit. Data collected by questionnaire included family demographics, medical histories of the child and mother, use of personal products, residential history, drinking water source, and physical activity (Hiatt et al. 2009). A fasting blood sample was collected at each visit; a urine sample and self-administered psychosocial questionnaire were collected once a year; and dietary recall interviews were conducted quarterly by telephone. The urine and blood samples were collected using protocols and materials provided by the Centers for Disease Control and Prevention (CDC). The biologic samples were collected for analysis of environmental exposure biomarkers, hormones, glucose, insulin, lipids, and genetic polymorphisms.

The GUF research team consisted of university-based researchers and community partners who were co-investigators actively engaged in the research as well as study staff. All members of the research team completed human subject research training as required by the CCHMC IRB.

The epidemiology study protocol was first approved by the CCHMC IRB in July 2004 and annually thereafter. The consent form specified that elevated blood pressure, blood sugar, insulin, and cholesterol would be reported to the parents of the study participants. The consent form did not specify the exposure biomarkers to be analyzed, include specific language about reporting these results to the study families, or include the option to decline receipt of test results. However, the consent form did state that “The investigators will tell you about significant new findings developed during the course of the research and new information that may affect your health, welfare or willingness to stay in this study.” No Certificate of Confidentiality (COC) was in place in the early years of the study. Recently, a COC was received that retroactively covers the data collected since the inception of the study.

A pilot study was conducted in winter 2006 to determine whether biomarkers of exposure could be detected in the study girls. Samples obtained in the first year from 30 girls recruited from each of the three BCERC sites were included in the pilot study. The other sites included Mount Sinai School of Medicine (New York, NY) and Kaiser Permanente of northern California (several locations in the San Francisco Bay area). The urine and serum analyses were conducted by the Environmental Health Laboratory at the National Center for Environmental Health at the CDC. Classes of compounds were selected based on their biologic activity, relevance to pubertal development, and feasibility. Urine samples from all three cohorts were analyzed for compounds such as phenols, phthalates, phytoestrogens, and cotinine (Wolff et al. 2007). Blood samples from the northern California and Greater Cincinnati cohorts were analyzed for compounds such as metals, brominated flame retardants, polychlorinated biphenyls, and persistent pesticides. At the request of the CDC, additional analytes not known to be endocrine disruptors at that time, such as perfluorochemicals (PFCs), were included in the pilot study.

In late winter 2006, the CDC alerted our lead epidemiologist that there were elevated serum perfluorooctanoate (PFOA) levels among girls in the Greater Cincinnati cohort compared with the national median for older children (12–19 years of age) in the 1999–2000 National Health and Nutrition Examination Survey (NHANES), as measured by the same CDC laboratory.

PFOA is a common PFC, a class of man-made compounds extremely resistant to environmental degradation. Recent publications had reported its detection in the blood of humans (Emmett et al. 2006). On closer analysis, we determined that the elevated results belonged to study participants recruited from the northern Kentucky school district. We designated this district community B and the district north of the Ohio River in southwestern Ohio as community A. For the pilot study, the median PFOA value for community B (26.4 ng/mL) was significantly higher than for community A (7.0 ng/mL). The national median value for older children (12–19 years of age), based on the 1999–2000 NHANES, was 5.6 ng/mL (Calafat et al. 2007). Over the next 12 months, the research team sought to verify the results, investigate likely sources, identify possible health effects, and determine what to do with this unexpected finding.

## Methods

In spring–summer 2006, we focused our attention on the PFOA findings to determine what additional studies and follow-up should be conducted. Efforts to identify potential sources of exposure were inconclusive. We presented our preliminary findings to our BCERC colleagues and shared with them our concern about how to proceed.

In summer 2006, the research team convened two “biomarker summits.” Attendees included members of the research team as well as experts in exposure assessment and toxicology. The objectives included presentation of the pilot study results and environmental samples collected at the schools; identification of the IRB of record for the pilot study; establishing priorities for verifying the pilot study results; and developing the components of a communication strategy. We discussed possible target audiences, what results might be reported, and how we might report those results.

In fall 2006, serum samples from 15 of the original community B pilot study girls and from 30 additional girls from the same community were sent to the CDC. We wanted to determine whether the elevated PFOA concentrations persisted and whether other study girls in community B were affected. Although the repeated PFOA concentrations decreased between 2005 and 2006 in community B, serum levels of 88% of the girls were above the NHANES 95th percentile.

We continued to review the scientific literature for PFOA studies as well as lay media sources for information about PFOA contamination. The “Community Exposure to C8 [PFOA] in the Little Hocking Water Service Area Study,” taking place 250 miles up the Ohio River from Cincinnati, was particularly compelling (Emmett et al. 2006), as drinking water was determined to be the major source of exposure (compared with air), and the median PFOA blood concentration in children 6–10 years of age exceeded all other age groups except adults > 60 years of age. The U.S. Environmental Protection Agency regulators and researchers were contacted to learn about environmental sources and properties of PFOA as well as research addressing the biologic effects of exposure. Because of ongoing litigation and regulatory review, some of those contacted were nonresponsive. Online and print media were surveyed for information about known environmental sources, contamination patterns, and exposure avoidance recommendations. We also sought advice from the Silent Spring Institute (Newton, MA) and other researchers with practical experience reporting exposure biomarker results to study participants (Brody et al. 2007; Galvez et al. 2007).

As the decision to report the findings to the pilot study families evolved, the need for a communication plan became apparent. The plan needed to be responsive to all the target audiences, including study families, school administrators, health officials, and the media. The information needed to be factual, understandable, and consistent. It was important to use trusted individuals and institutions to communicate that plan (Galvez et al. 2007). The plan also needed to be comprehensive, anticipating questions and reactions that might arise.

The communication plan consisted of:

- PowerPoint presentation(s) tailored to different audiences
- Information packet with fact sheets from government web sites
- Graphs depicting the range of results for a sample analyte for each chemical class
- Graphs depicting the range of PFOA results from the pilot study and other references
- A one-page summary of preliminary GUF study findings
- Glossary of terms
- Frequently Asked Questions (FAQs) with answers
- A dedicated phone line for questions from study families
- A press release.

The CCHMC IRB was notified of all components of the communication plan. Members of the research team developed the FAQs to ensure that we were prepared to address questions likely to arise and that our responses were consistent no matter the respondent, setting, or target audience.

In May 2007, three family meetings were held in the two pilot study communities and at CCHMC. In community B, study families received a written invitation, and the study principal investigator (PI) phoned each family to encourage attendance. The study PI, an adolescent medicine physician known to all of the study families, presented a study update and the biomarker results using PowerPoint slides carefully developed by the research team. A template was designed that depicted the study data points for an analyte for each chemical class. For example, brominated diphenyl ether–100 served as the sample analyte for the brominated flames retardants. The 50th and 95th percentiles based on NHANES data recently reported by the CDC (Calafat et al. 2007) were included on each graph. Our consultations with Silent Spring Institute were especially helpful in the development of these graphics.

**Figure 1:**
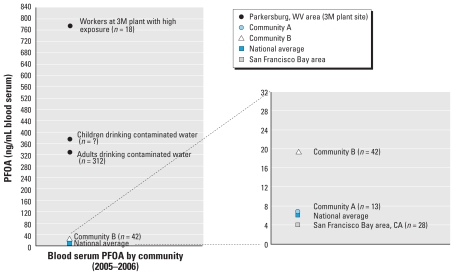
Relative exposure communication tool illustrating the differences in serum concentration of PFOA in occupational and related community populations, two BCERC cohorts, and the U.S. population; presented to Cincinnati BCERC Pilot Study Families, May 2007. Communities A and B and the San Francisco Bay area cohorts are all part of the BCERCs. Graphic on right is an expansion of selected data from graphic on left. Data from Emmett et al. (2006) and Calafat et al. (2007).

The PFOA findings were presented in a graphic that compared the results for communities A and B with data from the 1999–2000 NHANES national data set, a Parkersburg, West Virginia, cohort, and the San Francisco Bay area BCERC cohort. These additional data points were intended to provide context for the study families to interpret and compare the local data and their child’s results, as shown in Figure 1. We used the West Virginia data because Parkersburg is < 250 miles up the Ohio River from Cincinnati.

Each study family whose child participated in the pilot study received a report of the serum and urine biomarker results of their child. A sample analyte for each chemical class of biomarker was presented; the chemical classes included phenols, phthalates, cotinine, metals, phytoestrogens, brominated flame retardants, polychlorinated biphenyls, and persistent pesticides. Perfluorooctane sulfonate and PFOA were reported for the PFCs. The median for Greater Cincinnati (based on the 30 participants in communities A and B), national references (Calafat et al. 2007; National Center for Environmental Health 2005), and the child’s result were reported for each analyte.

The handout packet included a list of references, a glossary of > 70 terms, and copies of web site materials about the different biomarkers. These materials were downloaded from web sites maintained by federal agencies, state health agencies, and National Institutes of Health–sponsored research programs. The CCHMC IRB was notified of all materials provided to the study families.

At the meetings, time was allocated for questions and answers as well as one-on-one and small group discussions facilitated by the study PI and co-investigators. PFOA-related questions pertained to *a*) sources, pathways, and duration of exposure(s); *b*) possibility of follow-up studies that would include family members; *c*) likelihood of bioaccumulation; *d*) factors likely associated with higher concentrations, such as length of residence; *e*) recommendations for preventing future exposures; and *f*) implications for the puberty study. Given the dearth of specific information about exposure sources and pathways as well as health impacts, attendees asked why the information was being emphasized and should study families be concerned. Responses to these questions sought to allay concerns, emphasizing the absence of relevant data, the lack of standards, and the multiplicity of consumer products containing this compound.

In community B, the superintendent of the school district attended the meeting, having been alerted to the results by the study PI. The superintendent and several attendees commented that the findings might have implications beyond the study families and perhaps the county. The superintendent remarked that CCHMC is a trustworthy institution, inferring that there would be follow-up if health concerns came to light.

Three percent of the study families from community A and 41% of the study families from community B attended the meetings in their respective neighborhood school. Seventy-eight percent of the families attending the meetings completed a one-page evaluation of the program. Respondents generally felt the program met their expectations, that the information was presented in a manner they could understand, and that questions from the audience were answered satisfactorily. Respondents expressed interest in learning more about the study findings, both individual and aggregate results, especially from the psychosocial questionnaires as well as the exposure biomarkers, hormone analyses, and maturation assessments. As with the topics addressed in the question-and-answer period, respondents wanted more information about PFOA and ongoing updates. We mailed meeting-specific information packets to those study families who could not attend the meetings.

The UC Department of Public Relations and Communications had been alerted in advance about the family meetings. A press release was drafted should the need arise for a more public statement concerning the findings. Soon after the family meetings, members of the research team met with relevant water district personnel in Greater Cincinnati to notify them of the biomarker findings and to inquire about the availability of local water sample analyses for PFCs. Officials assisted us with information about their water intake sites along the Ohio River, purification technologies at each treatment facility, quality control standards, and the geographic distribution boundaries of each facility.

## Discussion

The research team grappled with the decision to communicate the unexpected PFOA findings to the study families. A number of competing and complementary perspectives were considered until consensus was reached.

### The transdisciplinary model

Transdisciplinary science is best described as the interactive work of scientists from multiple disciplines on a common problem with a common conceptual frame work, resulting in novel insights and approaches (Rosenfield 1992). The transdisciplinary structure of the BCERC allows for the integration of diverse scientific and community perspectives. The Cincinnati BCERC included environmental epidemiologists, an adolescent medicine physician, biologists, toxicologists and breast cancer advocates who had worked together since the development of the research proposal. This diverse group of individuals brought a variety of expertise, life experiences, and perspectives to the table:

- The researchers from the UC Department of Environmental Health had years of experience in reporting individual results to study participants from pediatric lead exposure studies, medical monitoring programs (Wones et al. 2009), and community-based participatory research. These were positive experiences that gave us a framework to design our reporting-back mechanisms.
- A clinical perspective reflected the Hippocratic Oath, “Do No Harm,” and some team members feared unnecessarily stressing the parents in this nonintervention study. These team members argued that it was not right for us to give parents information about a contaminant where the possible health effects of the source(s) or solution(s) to limiting exposure were unknown. At that time, there was no formalized communication plan to report back study results except elevated lipids, high blood pressure, and at-risk psychosocial indicators.
- The epidemiology members of the research team experienced in population-based studies had long ago adopted the terminology of study participants versus study subjects. The term “participant” reinforces individuality and argues against the notion that the terms are interchangeable. “Study participant” implies an active role in which individuals are engaged in an exchange of information, whereas “study subject” implies a passive role in which information is transferred in a single direction. This perspective reinforced the viewpoint that full disclosure to the study families was appropriate.
- The scientists at the CDC Environmental Health Laboratory where the analyses had been performed supported informing the families about these findings. Likewise, colleagues within the BCERC network encouraged us to discuss these high PFOA levels in community B.
- Ultimately, it was the breast cancer advocates who were a key factor influencing the decision of when and how to report results back. The advocate members of the research team spoke out on behalf of the study families. They espoused the concept of a partnership with the study parents and reasoned that individuals respect honesty. This perspective as a stand-in or proxy parent of the girls argued for full disclosure of available information, even if the source(s) of the exposure and the health implications were unknown. They also considered the study participants and any pregnant family members as vulnerable populations because of their increased sensitivity to an increased body burden due to environmental exposures (Tamburlini et al. 2002).

### The precautionary principle and the right to know

At the time of our deliberations, the health consequences of PFOA were poorly understood, no health standard(s) existed, and it was unknown whether blood levels reflected past or current exposures. Our center was at a loss for recommendations as to how to remove this chemical from the body. The precautionary principle encourages policies that protect human health in the face of uncertain risks. The precautionary principle states that evidence of harm, rather than definitive proof of harm, should prompt policy action, and that the burden of proof should lie with manufacturers to demonstrate that chemicals are safe, rather than with the public to show that they cause harm (Kriebel et al. 2001). We used this principle to guide us as we took responsibility to communicate with our study families. We had to overcome the fact that we did not have all of the answers, but it was our responsibility to give study families the information we knew at that time, so that they could make informed decisions about their health and the health of their families. It was difficult to recommend any interventions to the parents of our study participants. The data suggested an air or water pathway, rather than product use, because the girls with elevated values were limited to one geographic area. At the time of the parent meetings, we did not know whether the exposure still existed, and we explained these gaps in our knowledge to them. We did suggest the use of filtered water, if they had a concern.

The principles of respect for persons, beneficence, and justice shape the conduct of research with human subjects (Fernandez et al. 2003; National Commission for the Protection of Human Subjects of Biomedical and Behavioral Research 1979). Right-to-know regulations addressing exposures of workers in workplace settings and residents in community settings provided guidance for sharing available (although sometimes incomplete) information. Study participants want to know their results and are naturally curious about their environmental exposures (Deck and Kosatsky 1999). Study participants deserve basic information about the risks of widely used chemicals that they may be exposed to at work, at home, or through their food and water (Morello-Frosch et al. 2009).

### Potential fallout from reporting results

Other factors were discussed as reasons not to share the PFOA results to study families at that time:

- Study recruitment was ongoing, and there was concern that these unexpected results could negatively influence enrollment and/or retention of the girls already enrolled in the study.
- Ongoing litigation and regulatory review of manufacturing facilities along the Ohio River that had disposed of PFOA into the environment concerned the research team. Public release of the biomarker findings might lead to Freedom of Information Act requests from news media or participants involved in legal actions at PFOA contamination sites around the country. Members of the research team were concerned about the possibility of legal challenges that might compromise the anonymity and confidentiality of study families. At the time, no COC existed for this study.
- Accurately reporting these findings to study families was going to be an extremely time-consuming task on top of existing day-to-day study commitments. There were no extra funds or support to pay for additional items such as retesting serum samples, testing water samples, or hiring support staff.
- PFOA biomarkers were not part of the original BCERC protocol. Because at the initiation of the pilot study PFCs were not biomarkers of interest in relation to puberty or breast cancer, some argued that we had no obligation to become involved in something that was outside our study objectives. Because these results had not been published in a peer-reviewed journal, there was some reticence to release the data.
- Some team members argued that we were not obligated to remedy the situation simply because we had encountered an unexplained phenomenon (Resnik 2009).

## Conclusions

Human research protocols must adapt as scientific methods improve, research standards evolve, new collaborations with diverse stakeholders develop, and community knowledge and expectations change. Our experience offers an example of communicating an unexpected research finding to the families of study participants. Although we have not yet developed a comprehensive protocol for communicating individual results, we have learned a number of important lessons for the future.

A realistic assessment of what should and can be shared needs to be addressed before recruitment begins. The consent form is a crucial document that must be developed with care, clearly stating what, when, and how aggregated and individual data will be communicated to whom. The option to opt out of receiving all or some of the study results can be included in the consent form to accommodate the preferences of individual participants. A COC should be acquired for the study, because it provides protection for study participants beyond that provided by the process of obtaining informed consent.

Consider forming an advisory committee of stakeholders, including study participants. This venue can serve to inform the research team about expectations of key stakeholders vis-à-vis the study and changes in public knowledge and opinions about environmental exposures and health. It is also an opportunity for researchers to educate key stakeholders about research, its strengths, its uncertainties, and the prolonged time frame.

A good transdisciplinary research team will by its very nature reflect a diversity of perspectives. Members can have strong opinions reflective of their training and experiences, leading to vigorous disagreements as to how to proceed. Resolution of issues can take many months as team members assimilate new information and perhaps revise attitudes and approaches. When members are willing to listen to these diverse opinions and are open to learning and change, the dialogue can result in a solution that is acceptable to all and benefits the research team, study participants, and the general public.

With the transdisciplinary model becoming a frequent research design, all members of the research team should be able to inspect and review the data so challenging issues—such as whether, when, and how to communicate research findings—can be discussed openly and candidly among all members. Community partners, like university-based researchers and study staff, should complete all the standard training for human subject research so the breadth of experience, knowledge, and perspectives of all the members of the transdisciplinary team can be accessed. It is our experience that those community members will drive the “Right to Know” principles and encourage open and timely communication when the research involves human participants.
